# Matrix stiffness modulates the activity of MMP-9 and TIMP-1 in hepatic stellate cells to perpetuate fibrosis

**DOI:** 10.1038/s41598-019-43759-6

**Published:** 2019-05-13

**Authors:** Dariusz Lachowski, Ernesto Cortes, Alistair Rice, David Pinato, Krista Rombouts, Armando del Rio Hernandez

**Affiliations:** 1 Cellular and Molecular Biomechanics Laboratory, Department of Bioengineering, Faculty of Engineering, Imperial College London, South Kensington Campus, London, SW7 2AZ UK; 2 Hammersmith Hospital, Imperial College London, London, W12 0HS UK; 3 Regenerative Medicine and Fibrosis Group, Institute for Liver and Digestive Health, University College London, Royal Free Hospital, London, UK

**Keywords:** Cancer models, Cell biology

## Abstract

Liver fibrosis is characterised by a dense and highly cross-linked extracellular matrix (ECM) which promotes progression of diseases such as hepatocellular carcinoma. The fibrotic microenvironment is characterised by an increased stiffness, with rigidity associated with disease progression. External stiffness is known to promote hepatic stellate cell (HSC) activation through mechanotransduction, leading to increased secretion of ECM components. HSCs are key effector cells which maintain the composition of the ECM in health and disease, not only by regulating secretion of ECM proteins such as collagen, but also ECM-degrading enzymes called matrix metalloproteinases (MMPs) and their inhibitors (TIMPs). Uninhibited MMPs degrade ECM proteins to reduce external rigidity. Using fibronectin-coated polyacrylamide gels to alter substrate rigidity without altering ligand density, we show that fibrotic rigidities downregulate MMP-9 expression and secretion, and also upregulate secretion of TIMP-1, though not its expression. Using tissue immunofluorescence studies, we also report that the expression of MMP-9 is significantly decreased in activated HSCs in fibrotic tissues associated with hepatocellular carcinoma. This suggests the presence of a mechanical network that allows HSCs to maintain a fibrotic ECM, with external rigidity providing feedback which affects MMP-9 and TIMP-1 secretion, which may become dysregulated in fibrosis.

## Introduction

Liver fibrosis in hepatocellular carcinoma (HCC) is characterised by a remarkable extracellular matrix (ECM) stiffness, with extensive deposition and cross-linking of extracellular proteins, including fibrillar and basement membrane collagens. These proteins are primarily secreted by activated hepatic stellate cells (HSCs), myofibroblast-like cells that remodel the extracellular matrix and drive fibrosis in a disease state^1,2^. In healthy liver tissue, hepatic stellate cells reside in a quiescent state with cytoplasmic vitamin A-rich droplets, a low number of mitochondria, and a distinct rough endoplasmic reticulum. Following liver injury, HSCs become activated, and in addition to increased production of collagen and other ECM proteins, lose their vitamin A-rich droplets and become increasingly contractile and proliferative. Activated HSCs are also associated with increased inflammatory signalling and altered matrix degradation, all of which can contribute to perpetuation of fibrosis^3,4^.

HSCs have a key role in remodelling and maintaining the ECM, and achieve this through secretion of ECM proteins such as collagen, as well as ECM-degrading enzymes known as matrix metalloproteinases (MMPs). MMPs are calcium-dependent zinc-containing peptidases and are responsible for the degradation and turnover of most components in the ECM, including collagen^5^. Quiescent HSCs maintain ECM homeostasis by balancing the extracellular proteolytic activity of MMPs with the production of ECM proteins, but when HSCs are activated in disease or following injury, excess collagen production and altered matrix degradation leads to a stiff fibrotic state^6^. In fibrosis resolution, increased activity of MMPs leads to collagen degradation and ECM softening, with consequent reversion of activated HSCs to their quiescent phenotype. Conversely, perpetuation of the stiff fibrotic state occurs when characteristics of the environment, such as altered stiffness, amplify the activated HSC phenotype^3,4^.

MMP activity can be regulated at multiple levels, such as transcription, translation, and regulation of proenzyme activation^7^. Additionally, cells can secrete tissue inhibitors of matrix metalloproteinases (TIMPs) that inhibit the activity of MMPs outside the cell. Different MMPs are inhibited by different TIMPs in a complex interaction network and therefore the specific balance of MMPs with their cognate TIMPs dictates the activity of the extracellular MMP pool. For example, TIMP-1 reacts with the zymogen form of MMP-9^8^. TIMPs bind to MMPs through both their N and C terminal domains. The N-terminal domain of TIMPs leads to MMP inhibition by chelating the zinc ion in the active site of MMPs. The C terminal domain binds with high affinity to the haemopexin domains of MMPs, and this interaction is likely to be inhibitory^9^.

The pathological state of fibrosis is associated with an increased matrix stiffness due to the altered composition of the ECM, with stiffness dependent on both collagen abundance and cross-linking^10^. This increased rigidity is detected by HSCs through cell surface receptors known as integrins, leading to HSC activation^11–13^. HSC-mediated remodelling is a key process in ECM homeostasis, and it is known that the ECM can regulate its own composition through biochemical regulation of the secretion profile of resident fibroblasts^14^. Since many signalling pathways in HSCs have been shown to be highly mechanosensitive, e.g. rigidity inhibiting the HNF4α transcriptional network^11^ or integrin-mediated activation of YAP^13^, it is likely that external rigidity from fibrosis may affect HSC-mediated ECM remodelling at the protein expression and secretion levels. The matrix remodelling proteins matrix metalloproteinase 2 (MMP-2) and 9 (MMP-9) are two key MMPs secreted from HSCs that degrade collagen^15^, and a sensitivity of MMP and TIMP secretion by HSCs to external rigidity may underlie an important feedback loop that regulates the composition of the ECM in fibrosis.

To investigate the role of matrix stiffness on the function of MMP-2, MMP-9 and TIMP-1, we cultured HSCs on substrates of varying rigidity (4, 12 and 25 kPa), mimicking healthy and fibrotic liver tissue stiffnesses^16^. We then assayed changes in mRNA and protein expression and secretion of MMP-2, MMP-9, and TIMP-1 in response to external stiffness to analyse the possible mechanotransduction network that surrounds MMP-mediated ECM homeostasis. We have tested the hypothesis that the rigid environment created by fibrosis can modulate the expression and secretion of proteins, specifically MMP-9 and TIMP-1, to alter the ECM in either resolution or perpetuation of fibrosis. Our results indicate that rigidity promotes the perpetuation of fibrosis as external stiffness downregulates the activity of MMP-9.

## Results

### Substrate rigidity inhibits MMP-9 gene expression

Fibrosis in the liver is associated with specific rigidities, as determined through ultrasound based transient elastography. Liver stiffness values below 6 kPa are designated as normal, with values around 8–12 kPa designated as advanced fibrosis and cirrhosis. Further disease progressions including portal hypertension and esophageal varices have been shown to increase the stiffness to greater than 20 kPa^16^.

We hypothesized that substrate stiffness would affect MMP-2, MMP-9 and TIMP-1 gene expression. To alter substrate stiffness without altering ligand density, we fabricated fibronectin-coated polyacrylamide substrates, where rigidity could be tuned by altering the ratio of acrylamide to bis-acrylamide. Since we coated these substrates with the same amount of fibronectin, the ligand density was constant across samples so only the effect of rigidity was observed. We chose 4, 12, and 25 kPa rigidities to represent the progression from healthy to fully developed liver fibrosis, based on *in vivo* measurements^16^. HSCs were cultured on these polyacrylamide substrates of tuneable rigidity for 24 hours. We observed that HSCs became more elongated and less rounded with increasing rigidity (Supplementary Fig. 1).

**Figure 1:**
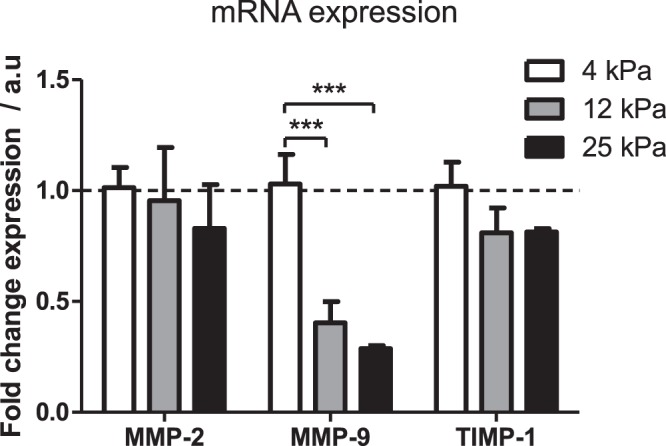
MMP-2, MMP-9 and TIMP-1 mRNA expression on different gel rigidities. mRNA expression of MMP-2, MMP-9, and TIMP-1 was assayed by RT-qPCR, normalised to control GAPDH mRNA and presented relative to 4 kPa sample. Data obtained from 3 separate experiments (n = 3). Results are expressed as mean ± s.e.m. ***Represents t test, p < 0.001. Dashed line represent relative mRNA RT qPCR expression (normalized to GAPDH) value of 1.0.

After cell collection, we used reverse transcription quantitative PCR to assess the relative mRNA levels of the target genes (Fig. 1). GAPDH was chosen as a suitable housekeeping gene for normalization as its expression was unaffected by stiffness (Supplementary Fig. 2). We observed that the expression of MMP-2 remained unchanged across rigidities. Interestingly, we observed that on 12 and 25 kPa substrates, expression of MMP-9 was greatly reduced in comparison to the 4 kPa substrate, indicating that MMP-9 expression is sensitive to external rigidity through mechanotransduction. Similar to MMP-2, we observed that substrate stiffness did not significantly affect the TIMP-1 expression.

### Substrate rigidity inhibits intracellular protein levels of MMP-9 and TIMP-1

**Figure 2:**
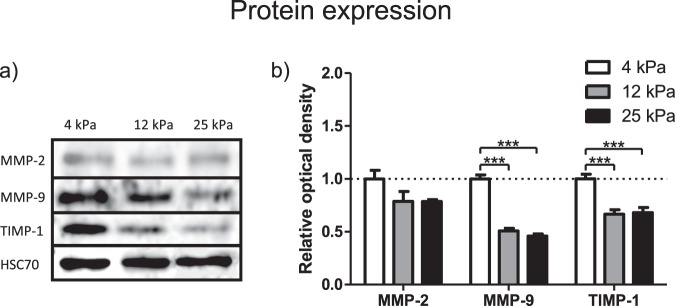
MMP-2, MMP-9 and TIMP-1 protein expression on different gel rigidities. (**a**) Protein expression of MMP-2, MMP-9 and TIMP-1 as assayed by Western Blot. HSC70 presented as control protein. (**b**) Optical density of Western Blot bands relative to 4 kPa sample. Data obtained from 3 separate experiments (n = 3). Results are expressed as mean ± s.e.m. ***Represents t test, p < 0.001.

The same experimental setup described in the previous section was used for Western Blot analysis, and performed to assess the intracellular protein expression of MMP-2, MMP-9 and TIMP-1 across different substrate rigidities (Fig. 2, Supplementary Fig. 3). Though all these proteins have extracellular roles, the mechanical sensitivity of their intracellular protein abundance would indicate the breadth of the mechanotransduction network, and its ability to affect multiple points in ECM homeostasis signalling.

The intracellular protein amount of MMP-2 was unchanged with increasing rigidity, equivalent to the trend observed in mRNA expression. Also, in agreement with mRNA expression, we observed a marked and significant decrease (around 50%) of intracellular MMP-9 protein as rigidity increased from 4 to 25 kPa. Intriguingly, we observed a significant decrease of 40% in the intracellular protein levels for TIMP-1 when rigidity was increased from 4 to 25 kPa, despite our observation that TIMP-1 mRNA levels were unresponsive to stiffness. This suggested to us a further mechanism by which TIMP-1 intracellular protein levels are regulated.

### Substrate rigidity modulates the activity of secreted MMP-9 and TIMP-1

**Figure 3:**
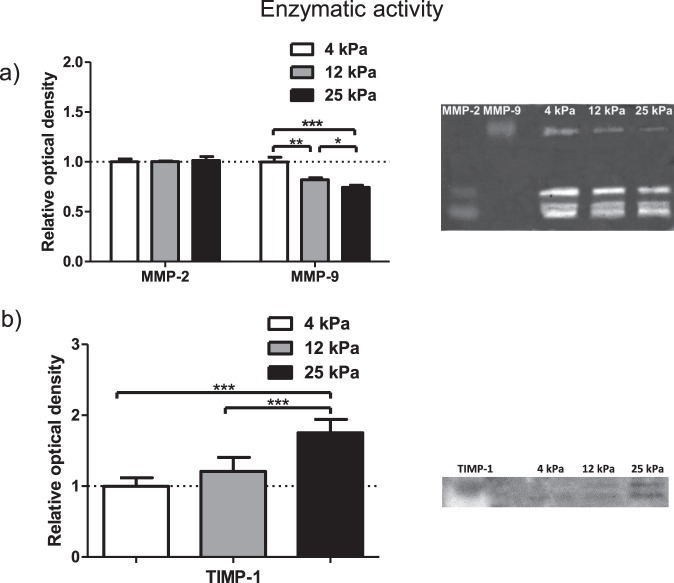
MMP-2, MMP-9 and TIMP-1 assayed activity on different gel rigidities. (**a**) Extracellular activity of MMP-2 and MMP-9 from HSC conditioned media assayed by gelatin zymography. Signal intensity of the bands presented relative to 4 kPa sample. Data obtained from 6 independent experiments. Results are expressed as mean ± s.e.m. *Represents t test, p < 0.05, **p < 0.01, ***p < 0.001. (**b**) TIMP-1 activity assayed by reverse gelatin zymography. Signal intensity of the bands presented relative to 4 kPa sample. Data obtained from 6 independent experiments. Results are expressed as mean ± s.e.m. ***Represents t test, p < 0.001.

To learn more about the effect of matrix rigidity on the target enzyme’s extracellular activity and to gain a more complete insight into the multi-level regulation of these proteins, we performed enzyme activity assays (Fig. 3, Supplementary Fig. 4). After 24 hours of culture on 4, 12 or 25 kPa substrates, cell culture medium was changed to serum free medium for the next 24 hours and then collected for further examination. This medium therefore contained any secreted MMPs and TIMPs. Gelatin zymography was performed to assay MMP-2 and MMP-9 activity, where band intensity represents level of degradation. The inhibitory activity of TIMP-1 on MMP-9 was assayed by reverse gelatin zymography, where band intensity represents level of TIMP-1 activity i.e. inhibition of degradation. Recombinant MMP-2 and MMP-9 were used in adjacent lanes to confirm the position of MMP-2 and MMP-9 mediated degradation within the gel.

MMP-2 activity was constant on each substrate, in line with levels of mRNA and protein expression, showing a lack of mechanosensitivity. MMP-9 activity significantly decreased with increasing rigidity, with the 25 kPa condition showing 25% less activity than the 4 kPa condition. These observations are consistent with the previous results indicating that rigid substrates decreased MMP-9 expression at both the mRNA and protein levels, and this trend is continued through secretion. Furthermore, we investigated TIMP-1 activity and observed that it was significantly increased for 12 and 25 kPa substrate-cultured cells. These findings contrast with the decreased levels of intracellular TIMP-1 when rigidity increases. This difference suggests that the lower intracellular levels of TIMP-1 seen at 12 and 25 kPa may be caused by the higher TIMP-1 secretion observed at these rigidities, which depletes the intracellular pool but enriches the extracellular pool of TIMP-1.

### TIMP-1 knockdown promotes extracellular MMP-9 activity

**Figure 4:**
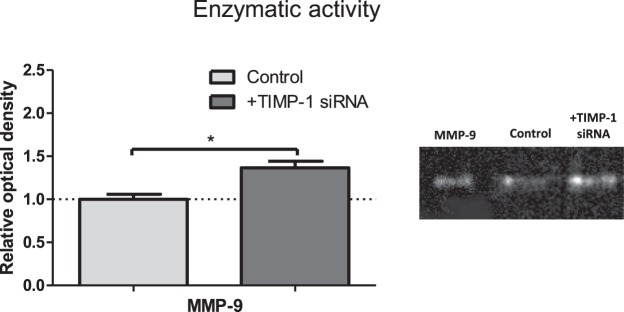
MMP-9 activity with TIMP-1 knockdown. MMP-9 zymography of HSC conditioned media from cell with or without TIMP-1 siRNA. Signal intensity of the bands presented relatively to untransfected control. Data obtained for 3 independent experiments. Results are expressed as mean ± s.e.m. *Represents t test, p < 0.05.

Since we observed TIMP-1 activity to increase in response to increased matrix rigidity using reverse zymography, we next quantitatively verified the relevance of this change in TIMP-1 activity with HSCs. We transfected HSCs with TIMP-1 siRNA to knockdown TIMP-1 (Supplementary Fig. 5), and assessed the enzyme activity of MMP-9, to indicate how TIMP-1 directly affects the MMP-9 activity of the cells. We observed a significant increase in MMP-9 activity of around 40% when TIMP-1 siRNA was used (Fig. 4), indicating that secreted TIMP-1 does indeed regulate MMP-9 activity in these cells. The large change in MMP-9 activity we saw emphasises the importance of our observations that levels of both MMP-9 and TIMP-1 are mechanosensitive.

### MMP-9 is downregulated in HCC

Hepatocellular carcinoma (HCC) is characterised by fibrosis, with high levels of extracellular matrix proteins. This fibrosis is maintained within the disease state, often through upregulation of ECM protein production. Although it is known that upregulation of MMP-9 occurs with progression from healthy liver to HCC^17^, the reported correlation between MMP-9 and HCC progression is related to the whole liver, and is not specific to HSCs. We therefore tested whether downregulation of MMPs occurs in HSCs in HCC, and would therefore promote the maintenance of the fibrotic stroma.

**Figure 5:**
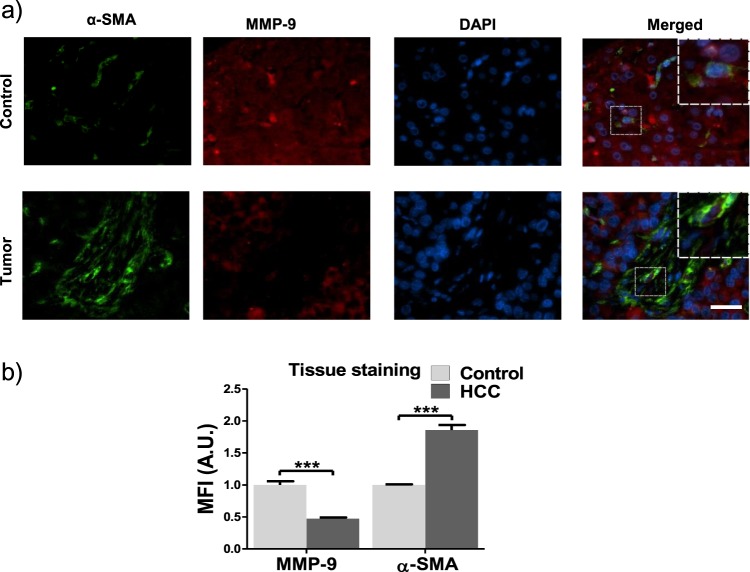
MMP-9 is downregulated in stromal tissues of HCC patients. (**a**) Representative images of tissue staining of stromal tissues from normal and HCC human patients, scale bar = 20 µm. Each image is a single slice as observed through immunofluorescence imaging. Inset represents magnification of area inside white square. (**b**) Quantification of MMP-9 and α-SMA (alpha smooth muscle actin) staining. Data was obtained from 20 HCC patients and 10 healthy patients. Bars represent mean ± s.e.m. ***Represents t test, p < 0.001.

We performed immunofluorescence staining on human tissues for control and HCC patients, using the activation marker α-SMA to identify activated stellate cells present within the tissue. Activated stellate cells were present in both healthy and HCC tissues, though at a lower level in healthy tissue (Fig. 5). This increased presence of α-SMA in HCC correlates with previous studies that link α-SMA expression to HCC progression^18^. We observed significantly lower levels of MMP-9 in activated HSCs in tissues from HCC patients than in healthy control patients, where the expression of MMP-9 in the activated HSCs present in HCC patients was almost negligible (Fig. 5). This indicates that one of the methods by which HCC may promote the disease state is through inhibition of MMP-9, which would otherwise degrade ECM components that contribute to fibrosis.

## Discussion

Remodelling of the extracellular matrix (ECM) is a common occurrence in many processes that occur in the healthy liver, including wound healing and morphogenesis, and includes the regulated secretion of proteases that degrade the components of the ECM^19^. In disease states, including fibrotic diseases such as hepatocellular carcinoma (HCC), stromal remodelling is deregulated and the altered ECM influences disease progression^20^. An understanding of the sensitivity of remodelling enzymes, such as matrix metalloproteinases (MMPs), to characteristics of this fibrotic ECM, such as rigidity, is of vital importance in understanding the disease^21^.

**Figure 6:**
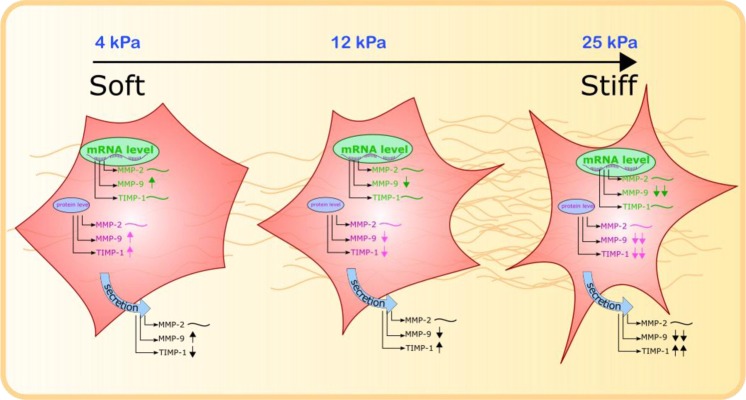
MMP-2, MMP-9 and TIMP-1 activity regulation on different gel rigidities. An illustration of matrix metalloproteinase 2 and 9 and tissue inhibitors of metalloproteinases 1 gene expression, protein expression and activity of secreted protein in cells cultured on different rigidity substrates. MMP-2 = matrix metalloproteinase 2, MMP-9 = matrix metalloproteinase 9, TIMP-1 = tissue inhibitor of matrix metalloproteinases 1. ~ represents no change in levels, ↑ and ↓ represent up and downregulation respectively.

We reveal that fibrotic matrix stiffness can decrease the activity of extracellular MMP-9 *in vitro*, and achieves this through downregulation of MMP-9 at the gene expression level. Furthermore, rigidity promotes secretion of TIMP-1, without affecting its gene expression (Fig. 6). This suggests a positive feedback loop in which the stiffness aspect of fibrosis, through mechanosignalling, promotes fibrosis perpetuation by inhibiting MMPs that catalyse ECM degradation. We observe evidence for this feedback loop at rigidities comparable to those in liver fibrosis^16^. The *in vivo* environment for HSCs is much more complex than our *in vitro* environment, where a multitude of biochemical and mechanical stimuli may promote or inhibit our proposed pro-fibrotic positive feedback loop. Our results are limited in that only the *in vitro* effect of matrix stiffness is analysed, and should therefore be combined with future studies which analyse the role of other aspects of fibrosis, such as changes in ECM architecture, to fully resolve the mechanisms surrounding MMP-9 and TIMP-1 regulation.

Though mechanisms that maintain MMP-9 and TIMP-1 levels in healthy homeostasis are not investigated here, we propose that in softer environments, the signalling pathways that modulate MMP-9 and TIMP-1 activity in response to high stiffness are not activated. Since we still observe MMP-9 expression at 25 kPa, albeit significantly reduced, this indicates that mechanotransduction of a soft substrate only partly promotes expression. *In vitro*, components of the cell culture medium likely promote a basal level of expression with further activation if the cell is on a soft environment. *In vivo*, the complex extracellular milieu also provides a wealth of possible signalling pathways promoting a basal level of expression, with external rigidity as an influencing factor.

Since we show that external rigidity alone affects MMP-9 and TIMP-1 activity, we demonstrate the importance of mechanotransduction of high rigidities in liver fibrosis, in concurrence with studies that link the rigidity of the fibrotic liver to disease progression^22^. By using an *in vitro* system whereby culture conditions only differ in substrate rigidity, any difference in phenotype can only be described as a product of mechanotransduction. Though cells can also sense a soft environment through mechanotransduction, our results indicate that the activity of remodelling enzymes is sensitive to external rigidity. Mechanosensing of rigid substrates plays an important role in the progression of other cancers such as pancreatic ductal adenocarcinoma^23^, as well as diseases such as cardiac hypertrophy and muscular dystrophy^24^. Therefore, the positive feedback system we observe, i.e. increasing rigidity inhibits expression of MMP-9, may underlie the perpetuation of fibrosis in the liver.

The understanding of this previously unobserved mechanical network may have therapeutic applications, as drugs could be used that target either MMPs or TIMPs, or the specific proteins that link the mechanical environment to MMP or TIMP regulation. Overexpression of MMP-9 has been seen in animal models of abdominal aortic aneurysms, and treatment with doxycycline has been shown to downregulate this expression^25^, suggesting the applicability of MMP-9 modulation in therapeutic approaches to other diseases. Therapeutics have also been developed against components of mechanosignalling networks^26^, and therefore our results could be applicable in guiding therapeutic development that seeks to reduce fibrosis in the liver, by targeting the mechanical network linking the fibrotic environment to the mechanosensitive proteins that can degrade it.

Our results reveal the mechanosensitivity of MMP-9 expression and secretion, as well as secretion of its inhibitor TIMP-1. This suggests a mechanosensitive feedback network in which the rigid environment of fibrosis can prevent its own degradation by downregulating the activity of a protease that would otherwise degrade it.

## Materials and Methods

### Tissues microarrays

Tissue microarray (TMA) blocks were constructed as previously described^27^ using 20 archival paraffin-embedded HCC tissue blocks and 10 healthy controls retrieved from the Imperial College London Tissue Bank (Ethical Approval nr.R15058). A consultant histopathologist reviewed all the materials prior to inclusion on freshly cut hematoxylin & eosin (H&E) slides. We constructed TMA blocks using an MTA-1 Microarrayer (Mitogen, UK) following H&E-slide guided microdissection of target tumour and surrounding non-tumorous areas. We obtained triplicates of 1 mm cores from separate central and peripheral areas of tumor and matching surrounding liver. Adequate sampling of target tissues was confirmed on a freshly cut H&E section from the recipient TMA block before downstream analysis

### Cell culture

Primary culture-activated human hepatic stellate cells (passage 3–6, HHStec #5300 – ScienCell) were cultured at 37 °C, 5% CO_2_ in culture medium containing DMEM/Nutrient Mixture F-12 Ham (D8437, Sigma), 10% FBS (10270-106, Gibco), 1% penicillin/streptomycin (Sigma Aldrich, USA) and 1% fungizone (15290-026, Gibco). For the polyacrylamide substrate experiments, HSCs were detached from culture flasks with trypsin and i) seeded on polyacrylamide gels with different rigidities for zymography (or immunofluorescence) staining; ii) transfected with TIMP-1 siRNA on glass for MMP-9 zymography. For this purpose, cells were transfected according to the manufacturer’s protocol with 2.2 pmoles of TIMP-1 siRNA using interferIN (409-10, Polyplus) 24 hours after seeding.

### Polyacrylamide substrate fabrication

Coverslips were dipped in 0.1 M NaOH and left to dry. Dried coverslips were coated with 4.0% (3-aminopropyl)triethoxysilane (281778, Sigma) and washed with dH_2_O. Coverslips were dried and transferred to 2.5% gluteraldehyde (G6257, Sigma)/PBS and incubated at room temperature for 30 minutes, twice washed in dH_2_0 and left to dry at room temperature. Polyacrylamide gels of 4, 12 and 25 kPa mimicking healthy and fibrotic liver stiffnesses^16^ were prepared according to the protocol adapted from Wen *et al*.^28^. Gel stiffness was varied by adding 29:1 acrylamide/*bis*-acrylamide to a final concentration ranging from 4.7–10%. A working solution of PBS, acrylamide/*bis-*acrylamide (29:1) 40% vol (A7802, Sigma), TEMED (T9281, Sigma) and 10% ammonium persulfate were mixed at concentrations to achieve varying gel stiffness. A small drop of this working solution was applied to activated coverslips which were placed face down on hydrophobic, dichlorodimethylsilane (440272, Sigma) treated glass microscope slides and left to polymerise at room temperature for 45 minutes. Gel-coated coverslips were removed and stored in PBS at 4 °C.

To allow polyacrylamide gels to be coated with ECM proteins, gels were functionalised to expose NHS groups. For functionalisation, polyacrylamide gels were washed with PBS and coated with 50 μL Sulfo-SANPAH (sulfosuccinimidyl 6-(4′-azido-2′-nitrophenylamino)hexanoate) (803332, Sigma) (5 mg/mL, PBS) solution per coverslip and activated with UV light for 10 minutes. Polyacrylamide gels were washed with PBS and coated with human plasma fibronectin (F8095, Sigma) (10 μg/mL, PBS) and incubated for 1.5 hr at room temperature. Gels were washed once with PBS and cells were seeded on gels in culture medium. The same procedure was used for large area microscope slide preparation for proteoanalysis and genetic analysis.

### Western blot

Cell lysates from cells cultured on polyacrylamide gels were prepared with radio immunoprecipitation assay (RIPA) buffer (89900, Thermo-Fisher) and a protease inhibitor cocktail (78440, Thermo-Fisher) for 10 minutes on ice. Lysates were sonicated and clarified by centrifugation at 9600 × G, at 4 °C for 10 minutes. Protein concentration was determined by DC protein assay (500-0116, Bio-Rad). Samples were separated by 12% SDS-polyacrylamide gel electrophoresis and transferred to a PVDF membrane. Membranes were blocked with 5% Bovine Serum albumin (BSA, A8022, Sigma) and 0.1% Tween-20(P1379, Sigma) in PBS for 30 minutes. Primary antibodies were prepared in blocking solution and incubated overnight at 4 °C (MMP-2 – sc-10736, Santa Cruz, MMP-9 – sc-10737, Santa Cruz, Timp-1 – sc-5538, Santa Cruz, HSC70 – sc-7298, Santa Cruz). The membrane was washed three times in 0.1% Tween-20/PBS and incubated with horseradish peroxidase (HRP) conjugated secondary antibodies for 1 hour. Following three washes in 0.1% Tween-20/PBS, membranes were developed using Luminata crescendo HRP substrate (WBLUR0100, Millipore) and Syngene GeneGnome. Band intensities were analysed via the band densitometry plugin in ImageJ.

### Quantitative PCR

Total RNA was extracted from cells cultured on polyacrylamide gels for 24 hours with RNeasy Mini Kit (74104, Qiagen), according to the “RNeasy mini quick start protocol”. RNA template was reversed transcribed into cDNA by High-Capacity RNA-to-cDNA kit (4387406, Thermo-Fisher) according to manufacturer’s instructions. Quantitative real-time PCR was performed on a StepOne Plus Real-Time PCR system (Applied Biosystems) using SYBR Green PCR Master Mix (4309155, Thermo-Fisher). Relative gene expression was analysed by the ΔCt method with 3 targets: MMP-2, MMP-9, TIMP-1, and final values were normalised using ΔCt for GAPDH gene mRNA as a value of 1.0. Primer sequences were as followed: MMP-2 (F) TCTCCTGACATTGACCTTGGC, (R) CAAGGTGCTGGCTGAGTAGATC; MMP-9 (F) TTGACAGCGACAAGAAGTGG, (R) GCCATTCACGTCGTCCTTAT; TIMP-1 (F) TCAACCAGACCACCTTATACCA, (R) ATCCGCAGACACTCCAT; GAPDH (F) ACAGTTGCCATGTAGACC, (R) TTTTTGGTTGAGCACAGG.

### Gelatin zymography and reverse zymography

The zymography resolving gel was prepared with 4.6 ml sterile distilled water, 2.7 ml 30% acrylamide, 2.5 ml 1.5 M Tris (pH 8.8), 100 µl 10%SDS, 285 µl 2.8 mg/ml gelatine (porcine skin type A gelatine, Sigma, G2500), 6 µl TEMED and 100 µl 10%APS to reach 8% acrylamide concentration. The reverse zymography gel was prepared by adding MMP-9 to the gel described before to reach 10 ng/ml concentration. Stacking gel was prepared with 3.4 ml sterile distilled water, 830 µl 30% acrylamide, 630 µl 1 M Tris (pH 6.8), 50 µl 10%SDS, 5 µl TEMED and 50 µl 10% APS. The conditioned medium was collected from HSCs cultured on polyacrylamide gels for 24 hours before replacing culture medium with serum free medium, DMEM/Nutrient Mixture F-12 Ham (D8437, Sigma) for a further 24 hours was added to non-reducing Laemmli buffer. Control marker samples for the zymography were prepared from recombinant human MMP-2 (Calbiochem, PF037) and recombinant human MMP-9 (Calbiochem, PF038) 10 ng/ml and 20 ng/ml respectively and for the reverse zymography natural human TIMP-1 (Abcam, ab157282) 10 ng/ml, then added to non-reducing Laemmli buffer.

Samples and standards were loaded onto the gelatine gel and run for 50 minutes at 200 V using Novex® Tris-Glycine SDS Running Buffer (10X) (Novex, cat. LC2675). Sample volumes were the same for all samples, taken from conditioned media from culture of the same number of cells. Then, the gel was carefully removed from the cassette and placed in an air tight container and washed with 2.5% (v/v) Triton X-100, in sterile distilled water 4 times for 15 minutes. The gel was developed with developing buffer (10 ml 1 M Tris (pH 7,5), 8 ml 5 M NaCl (Fisher Scientific, cat. BP358), 1 ml 1 M CaCl_2_ (Sigma, cat. C7902), 1.6 ml 2.5%Triton X-100 and 179.4 ml sterile distilled water). 20 ml developing buffer was added and incubated overnight at 37 °C. Coomasie blue staining solution was prepared with 0,5 g Brilliant Blue (Sigma, cat.27816-25G), 250 ml methanol, 100 ml acetic acid (Sigma, A6283-100 ml) and 150 ml sterile distilled water. After incubation, the gel was carefully transferred to a plastic box and stained with Coomasie blue for 1 hour, followed by decanting of the staining solution. The gel was rinsed with destaining solution containing 1.5 l methanol, 50 ml Formic acid (Sigma, cat. F0507) and 3.5 l sterile distilled water. After achieving the desired destaining grade, zymography = transparent, digested bands and blue background, reverse zymography = clear background and blue bands, the gel was photographed with a UVP Biospectrum 500 Imaging System. Band digestion intensity, representing potential MMPs activity, was calculated using the ImageJ densitometry plugin.

### Immunostaining

For α-SMA and MMP-9 staining, FFPE blocks were sectioned, deparaffinised and rehydrated as described previously^29^. Antigen retrieval was performed by incubating the sections in boiling citrate buffer (pH 6) for 45 minutes and cooled down at room temperature for 25 minutes. α-SMA antibody (Abcam, UK, ab7817, 1/50), MMP-9 (Santa Cruz Biotechnology, sc-10737, 1/50) and secondary goat anti-mouse AlexaFluor 488 (Life Technologies, Paisley, UK; A-11029, 1/200) and goat anti-rabbit AlexaFluor 546 (Life Technologies, Paisley, UK; A-11003, 1/200). Antibodies were used following the abcam TBS based staining protocol (https://www.abcam.com/protocols/immunostaining-paraffin-frozen-free-floating-protocol). Stained sections were mounted with ProLong® Gold Antifade with DAPI (Life Technologies, USA). Images from immunofluorescence staining were acquired using a Nikon Ti-e microscope. Mean fluorescence intensity was quantified using Fiji software by measuring the mean grey value in the MMP-9 channel for the randomly selected regions showing α-SMA positive staining. The values obtained from the different regions of the same tissue sections were averaged and treated as one experimental replicate. The final result was calculated for n = 20 HCC and 10 healthy patients per condition.

### Statistics

Results were analyzed using Prism software (GraphPad, La Jolla, CA,USA). A 2-tailed Student’s t test for unpaired data was used to calculate the difference between mean values, *p < 0.05, **p < 0.01, ***p < 0.001.

## Supplementary information

Supplementary Figures
