# Supplementary material for: Matrix stiffness modulates the activity of MMP-9 and TIMP-1 in hepatic stellate cells to perpetuate fibrosis
Source: Sci Rep. 2019 May 13;9:7299. doi: 10.1038/s41598-019-43759-6 (PMC6514003; doi:10.1038/s41598-019-43759-6)
Supplement: Supplementary file 1 — Supplementary Figures [file 41598_2019_43759_MOESM1_ESM.pdf]

# Matrix stiffness modulates the activity of MMP-9 and TIMP-1 in hepatic stellate cells to perpetuate fibrosis

Dariusz Lachowski[1], Ernesto Cortes[1], Alistair Rice[1], David Pinato[2], Krista Rombouts[3], *Armando del Rio Hernandez[1]

*[1] Cellular and Molecular Biomechanics Laboratory, Department of Bioengineering, Faculty of Engineering, Imperial College London, South Kensington Campus, London, SW7 2AZ, UK*

*[2]Hammersmith Hospital, Imperial College London, London, W12 0HS, UK*

*[3] Regenerative Medicine and Fibrosis Group, Institute for Liver and Digestive Health, University College London, Royal Free Hospital, London, UK*

**Corresponding Author: Armando E. del Río Hernández, email: a.del-rio-hernandez@imperial.ac.uk*

# Supplementary figure 1

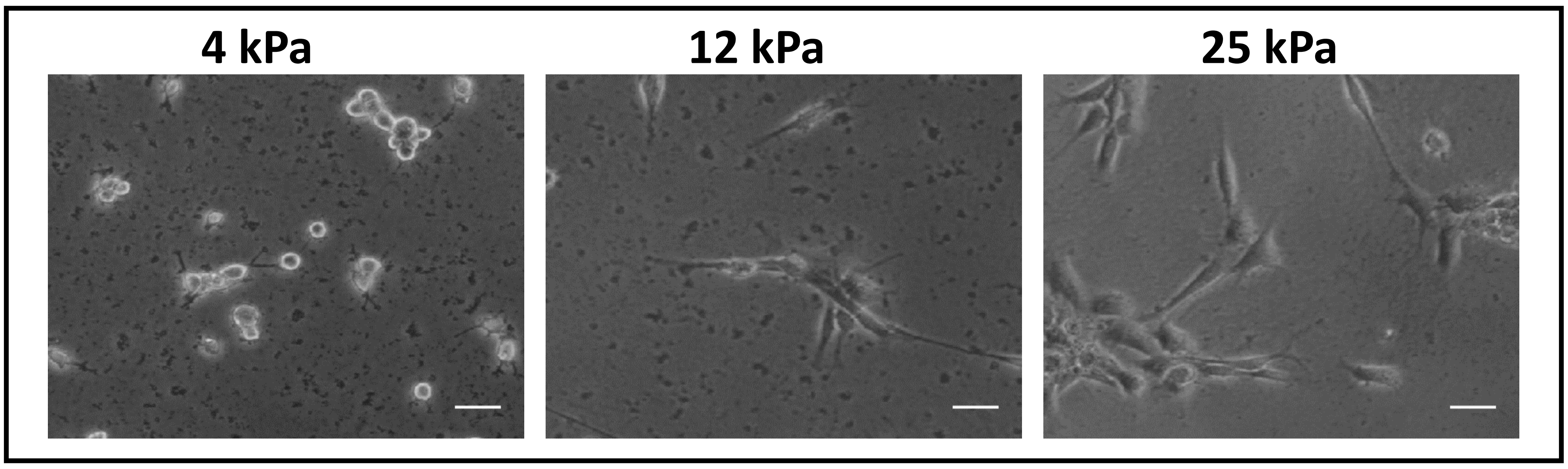


**Supplementary Figure 1. Changes in cell morphology are dependent on substrate stiffness.** Hepatic stellate cells were cultured on 4, 12 and 25 kPa substrates and after 24 hours the cell morphology was assessed.

## Supplementary figure 2

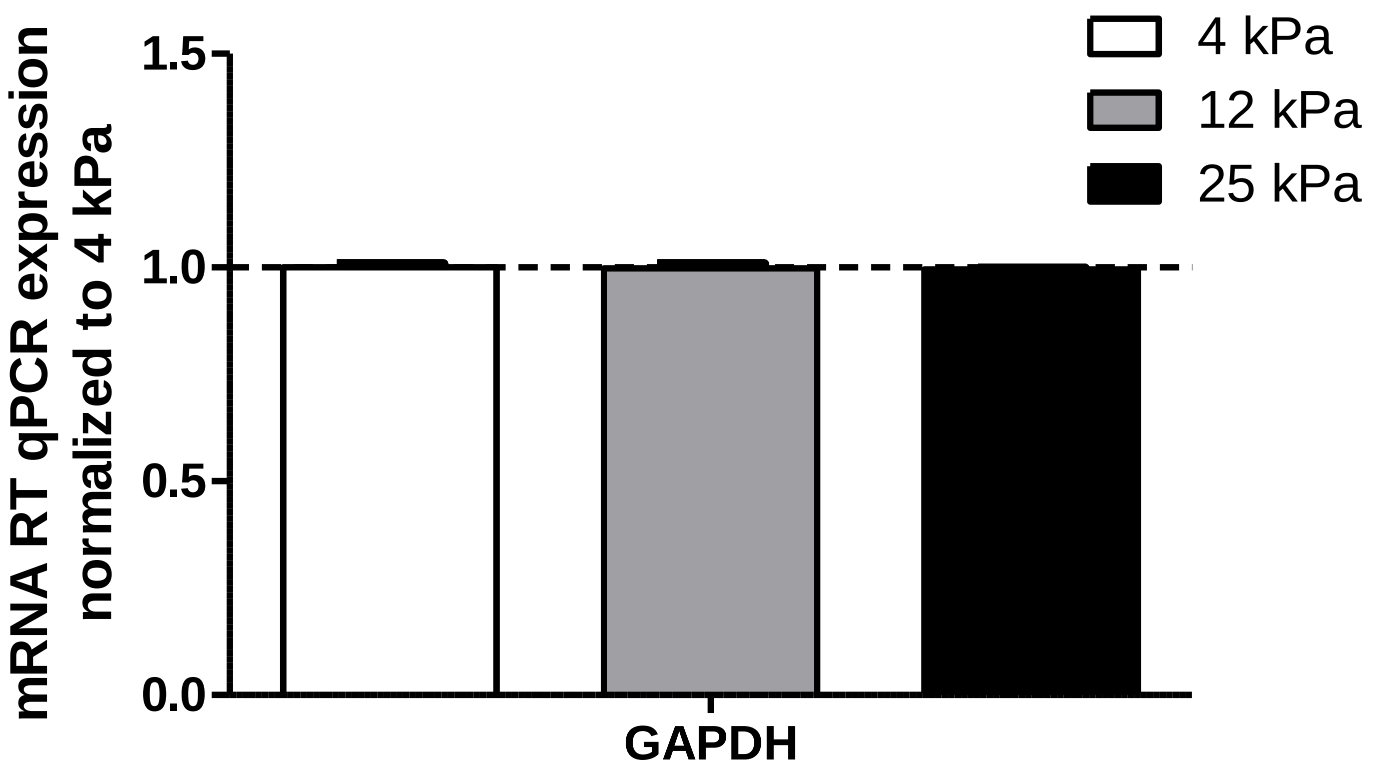


**Supplementary Figure 2. GAPDH mRNA expression in hepatic stellate cells does not change when cultured on different rigidity substrates**. mRNA expression of GAPDH was assayed by RT-qPCR, relative to 4 kPa. Data obtained from 3 separate experiments (n=3). Results are expressed as mean ± s.e.m. *** represents t test, $p < 0.001$

# Supplementary figure 3

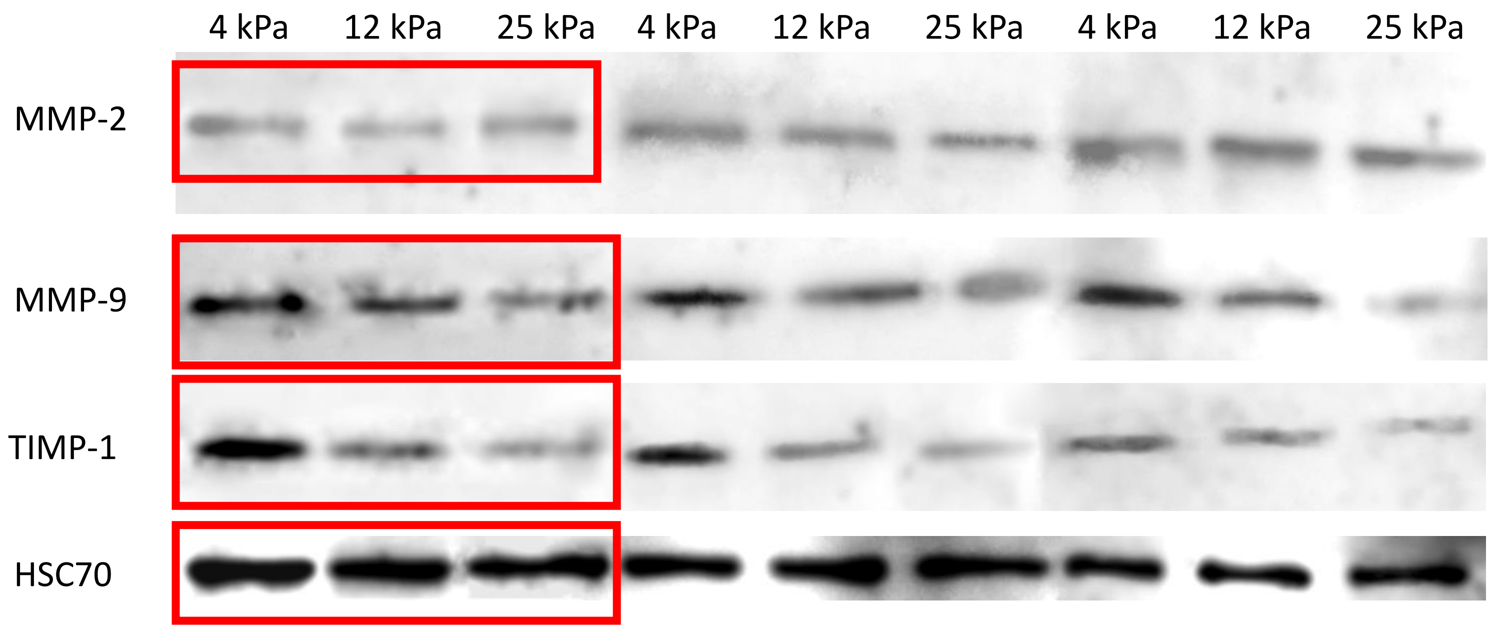


**Supplementary Figure 3. Western Blot analysis of protein expression in response to external rigidity**
Full Western blot membrane images for MMP-2, MMP-9, TIMP-1 and loading control HSC70. Rectangle marks the representative bands.

# Supplementary figure 4

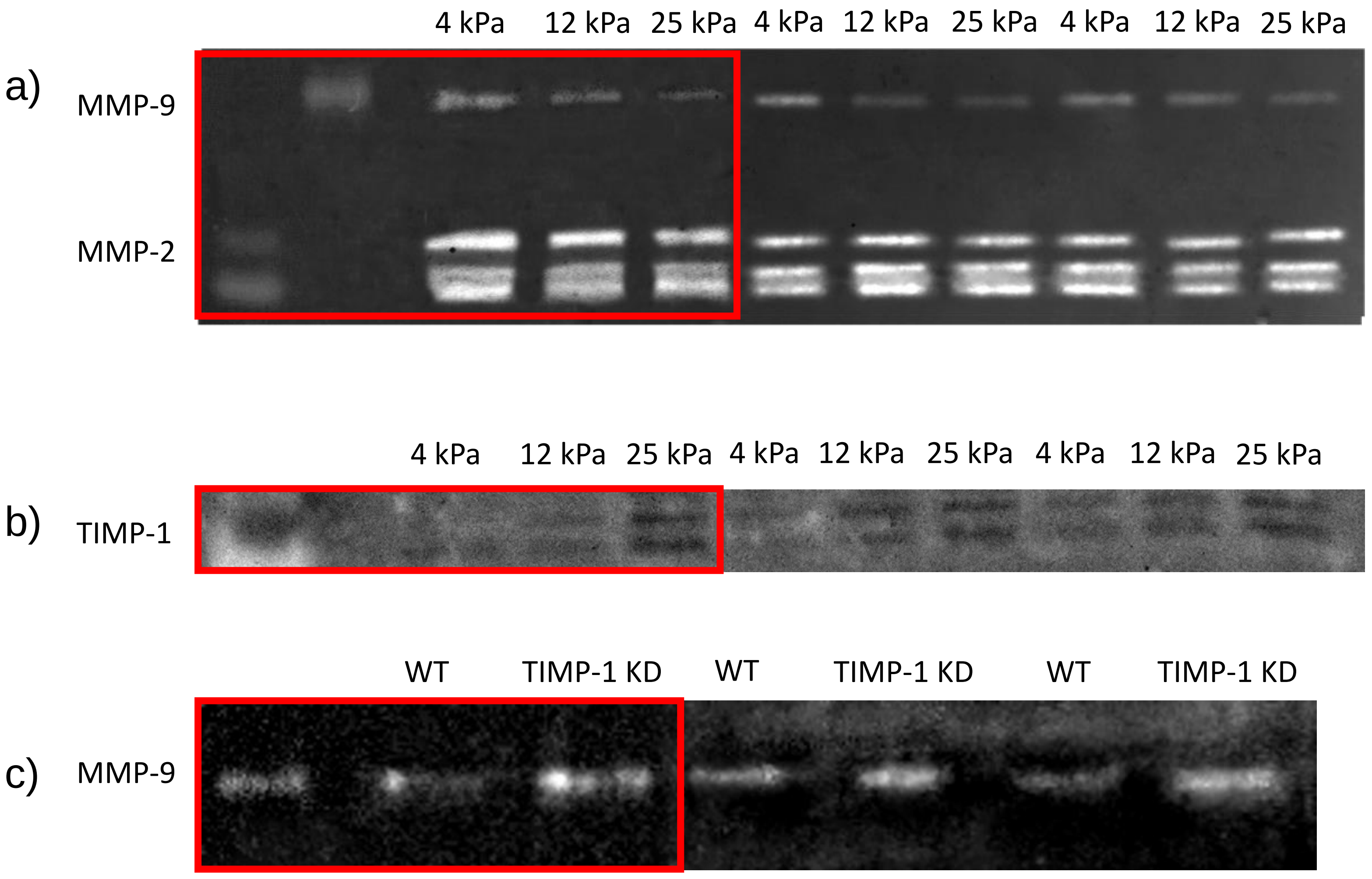


**Supplementary Figure 4. Enzymatic activity of MMP-9, MMP-2 and TIMP-1 in response to external rigidity assessed by zymography and reverse zymography.** Representative images of **a)** MMP-2 and MMP-9 zymography, **b)** reverse zymography of TIMP-1 in wildtype cells and **c)** MMP-9 zymography of TIMP-1 knockdown cells. Rectangle marks the representative bands.

# Supplementary figure 5

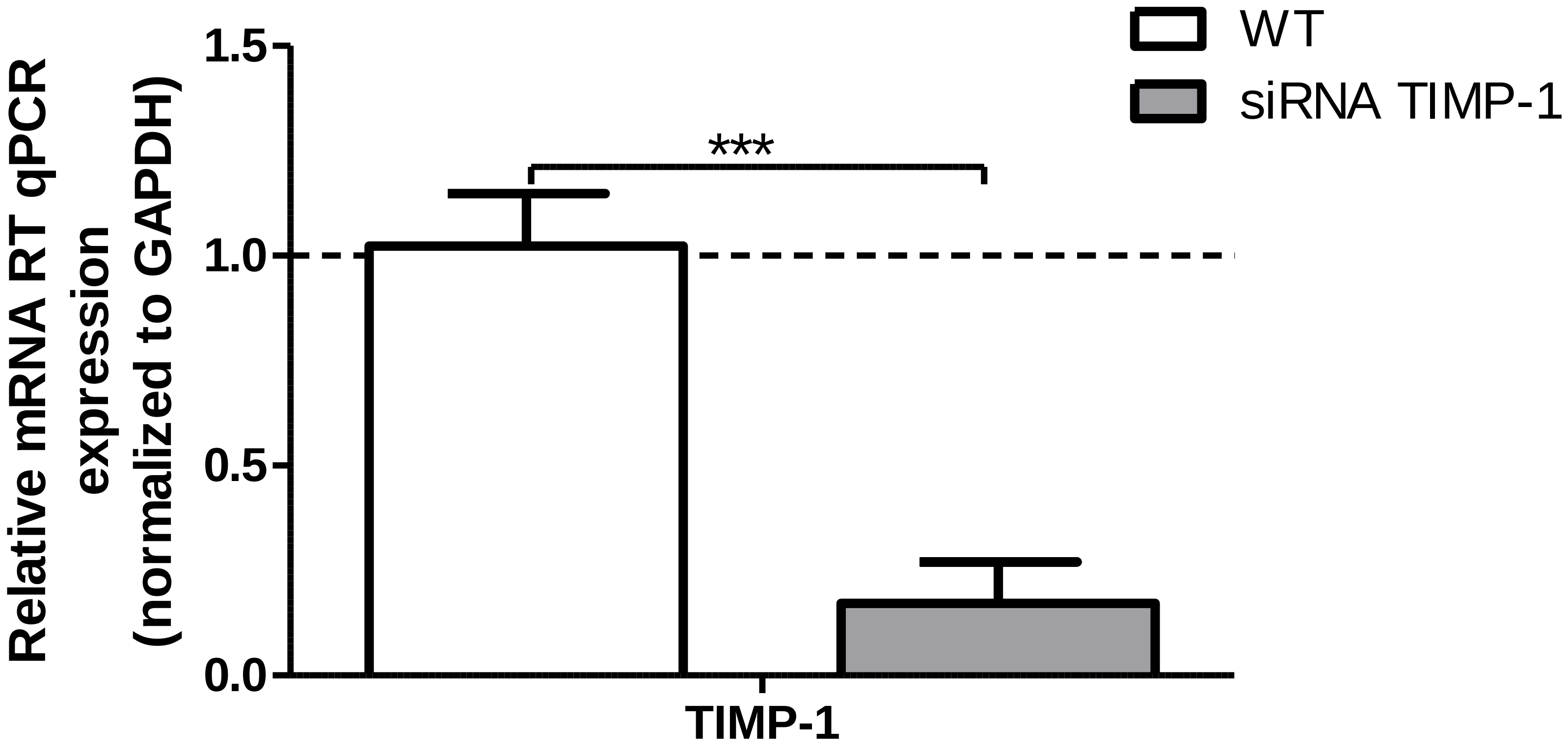


**Supplementary Figure 5 TIMP-1 mRNA expression in cells treated with TIMP-1 siRNA**.
mRNA expression of TIMP-1 was assayed by RT-qPCR, normalised to control GAPDH mRNA and presented relative to wildtype. Data obtained from 3 separate experiments (n=3). Results are expressed as mean ± s.e.m. *** represents t test, $p < 0.001$
